# P-1336. The in vitro activity of aztreonam-avibactam against KPC-positive and/or ceftazidime-avibactam-resistant Enterobacterales, ATLAS 2019-2023

**DOI:** 10.1093/ofid/ofaf695.1524

**Published:** 2026-01-11

**Authors:** Mark Estabrook, Julie Dickson, Gregory Stone, Katherine Perez, Daniel F Sahm

**Affiliations:** IHMA, Schaumburg, IL; IHMA, Schaumburg, IL; Pfizer, Inc., Groton, Connecticut; Pfizer, Inc., Groton, Connecticut; IHMA, Schaumburg, IL

## Abstract

**Background:**

Aztreonam-avibactam (ATM-AVI) is a β-lactam/β-lactamase inhibitor combination to treat infections caused by Gram-negative organisms, particularly those carrying metallo-β-lactamases (MBLs) and other β-lactamases. Aztreonam is stable to hydrolysis by MBLs and avibactam inhibits Class A, C, and some Class D enzymes. We examined ATM-AVI activity against Enterobacterales isolates that were either positive for the Class A carbapenemase KPC or resistant to ceftazidime-avibactam among isolates collected as a part of the ATLAS global surveillance program (2019-2023).

**Figure:**
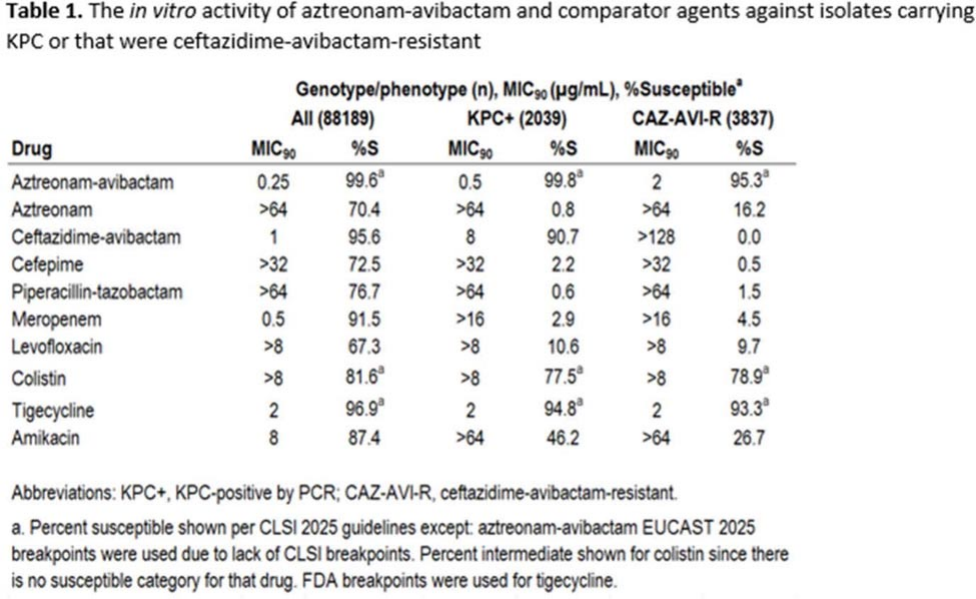

**Figure:**
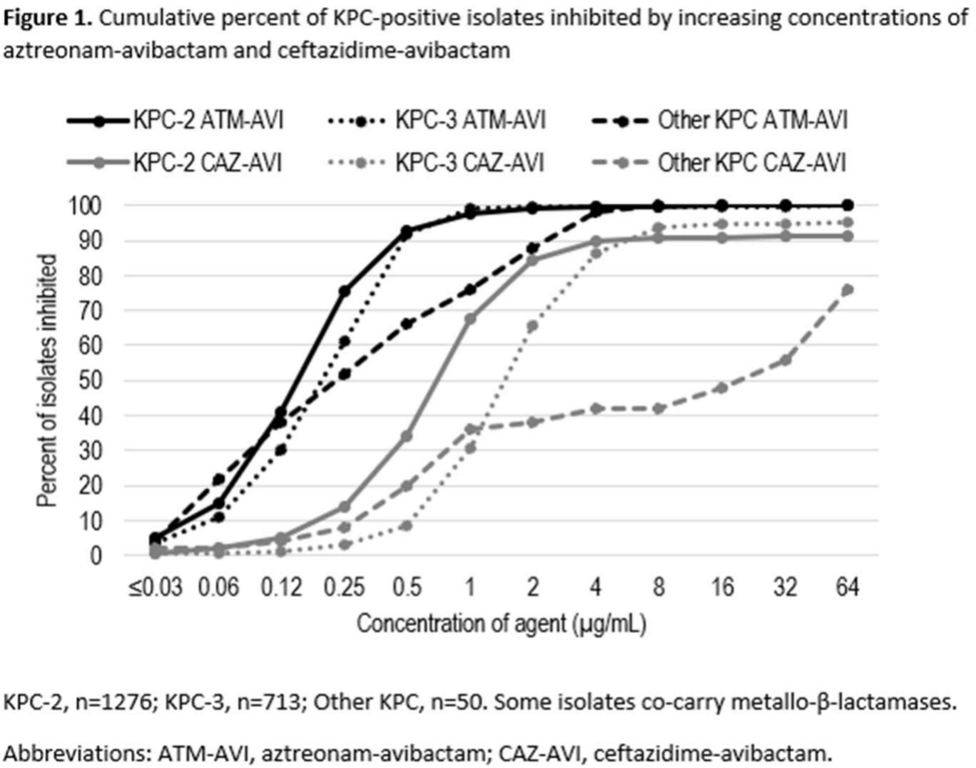

**Methods:**

88,189 isolates from 226 medical centers in 56 countries (excluding mainland China, Canada, and the USA) were collected and tested for susceptibility using the broth microdilution method according to CLSI guidelines. Analysis was performed with CLSI 2025 breakpoints. Isolates testing with meropenem MIC values >1 µg/mL or *Escherichia coli, Klebsiella pneumoniae, K. oxytoca,* or *Proteus mirabilis* isolates testing with ceftazidime and/or aztreonam MIC values >2 µg/mL were screened for β-lactamase genes by PCR.

**Results:**

Of the 88,189 isolates characterized, 2039 screened positive for KPC and 3837 were resistant to ceftazidime-avibactam. Against these, ATM-AVI *in vitro* activity was (genotype/phenotype, percent susceptible, MIC_90_): All, 99.6%, 0.25 µg/mL; KPC-positive, 99.8%, 0.5 µg/mL; ceftazidime-avibactam-resistant 95.3%, 2 µg/mL (Table 1). No comparator agents were active against this many isolates in any group. Of isolates carrying KPC, ≥98% were susceptible to ATM-AVI, while CAZ-AVI activity was 91% (KPC-2), 94% (KPC-3), and 42% (other KPC), noting that some of these isolates co-carried MBLs (Figure 1).

**Conclusion:**

Aztreonam-avibactam demonstrated potent *in vitro* activity against isolates that carry KPC carbapenemases and those that are ceftazidime-avibactam-resistant. While some uncommon variants of KPC can confer resistance to ceftazidime-avibactam, aztreonam-avibactam retained potency against these *in vitro*.

**Disclosures:**

Katherine Perez, PhD, Pfizer: Stocks/Bonds (Public Company)

